# Cardiovascular Risk Factors for Incident Hypertension in the Prehypertensive Population

**DOI:** 10.4178/epih/e2010003

**Published:** 2010-05-01

**Authors:** Soo Jeong Kim, Jakyoung Lee, Sun Ha Jee, Chung Mo Nam, Kihong Chun, Il Soo Park, Soon Young Lee

**Affiliations:** 1 Department of Occupational and Environmental Medicine, Ajou University School of Medicine, Suwon, Korea.; 2 Graduate School of Public Health, Yonsei University, Seoul, Korea.; 3 Institute for Health Promotion, Yonsei University, Seoul, Korea.; 4 Department of Preventive Medicine, Yonsei University College of Medicine, Seoul, Korea.; 5 Department of Preventive Medicine and Public Health, Ajou University School of Medicine, Suwon, Korea.; 6 National Health Insurance Corporation, Seoul, Korea.

**Keywords:** Hypertension, Progression, Cardiovascular disease, Health behavior, Prospective study

## Abstract

**OBJECTIVES:**

The aim of this study was to investigate the effect of changes in cardiovascular disease (CVD) risk factors on progression from prehypertension (PreHTN) to hypertension (HTN) using an 8-yr prospective Korean Cancer Prevention Study (KCPS) by the National Health Insurance Corporation (NHIC) in Korea.

**METHODS:**

A total of 16,229 subjects, aged 30 to 54, with new onset preHTN at baseline (1994-1996) in a biennial national medical exam were selected and followed up till 2004 at 2-yr intervals. All subjects underwent a biennial health examination including biochemical measurements and behavior. The log-rank test was performed to assess the relationship between changes in CVD risk factors and progression to HTN. The Cox proportional hazard model was used to identify factors influencing progression to HTN.

**RESULTS:**

With regards the progression rate in men, ex-smokers (42.9%), abstainers (37.5%), and regular exercisers (37.6%) showed a slower progression rate than continuous smokers (49.5%) and continuous drinkers (50.9%). In women, those who participated in regular exercise (22.6%) had a lower rate of progression than continuous non-exercisers (36.1%). According to the results of the Cox proportional hazard model, improvements in smoking (hazard ratio [HR], 0.756), drinking (HR, 0.669), regular exercise (HR, 0.653), body mass index (HR, 0.715), and total cholesterol (HR, 0.788) played a protective role in progression to HTN in men, while in women, participating in regular exercise (HR, 0.534) was beneficial.

**CONCLUSION:**

Improvements in CVD-related behaviors diminished the progression rate of HTN. This study suggests that individuals with PreHTN should be targeted for specific health behavioral intervention to prevent the progression of HTN.

## INTRODUCTION

Hypertension (HTN) is defined as a repeatedly elevated blood pressure (BP) exceeding a systolic blood pressure (SBP) over 140 mmHg and/or a diastolic blood pressure (DBP) over 90 mmHg [1]. It is clear that HTN has become a worldwide epidemic and an important risk factor of cardiovascular disease (CVD), which might cause fatal results in an individual such as stroke, myocardial infarction, ischemic heart disease, kidney disease, and peripheral vascular disease [1-4]. Revised reports have been published every four or five years since the Joint National Committee (JNC) on Prevention, Detection, Evaluation, and Treatment of High Blood Pressure was first established in 1977 [5]. In the recent JNC-7 report, a new category known as prehypertension (PreHTN) was added. In this category, the normal and high-normal BP of the JNC-6 criteria are combined because even normal BP (SBP 120 to 129 mmHg or DBP 80 to 84 mmHg) are potentially related to future CVD. Several studies have investigated the short-term and long-term progression rates from the non-hypertensive category to HTN [6-12]. According to the Framingham Heart Study, at the age of 55 yr, prehypertensive people had a two times higher risk of progression to HTN than normotensive people [8]. Many studies have suggested that lifestyle modifications might help people to manage HTN [6, 9, 10, 13]. Researchers have been interested in behavioral changes such as exercise, a healthy diet, smoking cessation, etc. For example, a follow-up of the data from the British Health and Lifestyle Surveys for 7 yr showed that the relative risk (RR) (95% confidence intervals, CI) of subjects who were overweight at follow-up but not at baseline was 1.65 (1.33-2.14) and that the RR of subjects who were overweight at baseline but not at follow-up was 0.58 (0.34-1.01) compared with those who were not overweight at baseline or follow-up. Those who were smokers at follow-up had a higher risk (RR, 95% CI: 1.12, 0.94-1.34) of HTN than those who were non-smokers at baseline or follow-up [9]. However, there have been few studies on the association between CVD risk factors and the progression of HTN among people with PreHTN in Korea. The purpose of this study was thus to examine factors influencing progression using a prospective cohort dataset, the Korean Cancer Prevention Study (KCPS) from the National Health Insurance Corporation (NHIC).

## SUBJECTS AND METHODS

### Study population

**Figure 1:**
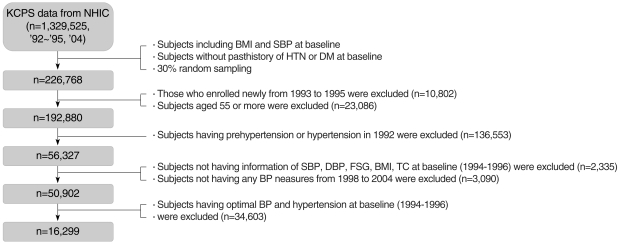
Data cleaning process for the study population. BMI, body mass index; SBP, systolic blood pressure; HTN, hypertension; DM, diabetes mellitus; DBP, diastolic blood pressure; FSG, fasting serum glucose; TC, total cholesterol.

NHIC provides a national health insurance program for government employees, private school teachers and their dependents. The NHIC data include the KCPS data from 1,329,525 Koreans between the ages of 30 and 95 yr who underwent biennial medical evaluations. A total of 784,870 (59.0%) were enrolled in 1992, 367,903 (27.7%) in 1993, 98,417 (7.4%) in 1994, and 78,335 (5.9%) in 1995. A more detailed description of the subjects can be found elsewhere [14, 15]. In the present study, after randomly selecting 30% of the subjects from the KCPS data, various exclusion processes were carried out (Figure 1). The baseline data were collected in 1994 and 1996. The data from participants were examined at baseline and at follow-up health examinations in 1998, 2000, 2002, and 2004. The study included examinees who underwent the biennial health examination at least once or more over the period of 8 yr. Participants were excluded if they had PreHTN or HTN in 1992. The final sample consisted of 16,299 subjects with PreHTN at baseline (1994-1996) who were followed every other year until 2004. The study protocol was approved by the Yonsei University College of Medicine ethical committee.

### BP measurements

The BP of participants was measured by registered nurses or trained technicians using a standard mercury sphygmomanometer or an automatic manometer. We used the mean BP of 1994 and 1996 to minimize misclassification error. The measured BP was classified as optimal (SBP <120 mmHg and DBP <80 mmHg), normal (SBP 120 to 129 mmHg or DBP 80 to 84 mmHg), high-normal (SBP 130-139 mmHg or DBP 85 to 89 mmHg), or HTN (SBP ≥140 mmHg or DBP ≥90 mmHg) based on JNC-6 criteria.

### Other measurements

Subjects were asked about their health behaviors and underwent biochemical measurements in each biennial health examination from 1992 to 2004. The health behavior characteristics included smoking, drinking, regular exercise and their family history of CVD-related diseases. Baseline data such as age and a family history of CVDs were based on the records from 1992. For smoking habits, the question asked was how many cigarettes he/she smoked. The respondents had the option of recording their current smoking status as never-smoker, ex-smoker or current smoker. Subjects were asked about their smoking habit status at each assessment to determine changes in smoking status. For drinking habits, the question asked was what are his/her drinking habits. The options were either "don't drink," "drink sometimes" or "drink often". Subjects were categorized into either non-drinker or current drinker (drinking sometimes and drinking often). Subjects were also asked whether they had changed their drinking status at the subsequent measurements. For regular exercise, the question asked was whether he/she was involved in regular exercise. The respondents replied either "yes" or "no" to the question. Subjects were classified into either those taking regular exercise or those not doing regular exercise. The information on smoking, drinking, and regular exercise at baseline was either from the data recorded in 1994 if available, or in 1996. For the biochemical measurements, the body mass index (BMI), serum glucose and total cholesterol were measured after fasting. The mean value of the 1994 and 1996 measurements for BMI, fasting serum glucose (FSG), and total serum cholesterol (TC) was used as the baseline. Changes in health habits and co-morbidity information were defined as any change between the data from 1994 or 1996 and the last follow-up data from 1998, 2000, 2002, and 2004.

### Statistical methods

The t-test and χ^2^-test were used to investigate the baseline characteristics of the study population according to the baseline BP. To assess the relationship between changes in CVD risk factors and progression to HTN, the log-rank test was performed. HRs and 95% CI were obtained by the Cox proportional hazard model to determine the impact of changes in CVD risk factors on progression to HTN. All statistical analyses were conducted using SAS version 9.1.3 (SAS Institute Inc., Cary, NC, USA).

## RESULTS

### Baseline characteristics of the study population according to baseline blood pressure

Table 1 shows the general characteristics of the study population according to baseline BP. The two groups (normal BP, and high-normal BP) differed significantly in all characteristics, except for the family history of CVD. The mean age was 38.6 yr for the normal BP group, and 39.8 yr for the high-normal BP group, respectively (p<0.0001). The high-normal group showed a significantly higher percentage of smoking, alcohol drinking and regular physical activity than the normal group. The high-normal group had an elevated mean SBP, DBP, FSG, and TC compared with the normal group (p<0.0001).

### Association between changes in cardiovascular disease risk factors and progression from prehypertension to hypertension

Tables 2, 3 summarize the results regarding the association between the changes in CVD risk factors and the progression rate in men and women. In men, the ex-smoker group (42.9%) showed a slower rate of progression compared to the continuous smoker group (49.5%) and the non-smoker group at baseline and the current smoker group at follow-up (51.0%). The progression rate in the continuously drinking group was found to be 50.9%, whereas that of the sober group stood at 37.5%; the rate in the non-drinking group at baseline and the drinking group at follow-up was 39.1%. Those participating in regular exercise had a slower rate of progression (37.6%) than that in those who did not take regular exercise (49.6%) and that in regular exercisers at baseline and non-regular exercisers at follow-up (49.3%). The consistently obese group showed a progression rate of 58.2% while the group in which weight fluctuated (normal weight to overweight and vice versa) demonstrated a 45.0% progression rate, resulting in a 13.2% difference. In terms of FSG measurements, the group with consistently normal FSG showed the lowest progression rate (47.3%). Unlike other CVD risk factors, the group with high FSG at baseline and normal FSG at follow-up (55.3%) and the group with normal FSG at baseline and high FSG at follow-up (52.1%) had a slower rate of progression than the consistently high FSG group (51.2%). In terms of TC measurements, the group with improvements in TC demonstrated lower progression rates than the consistently high TC group (Table 2). In women, smoking was excluded from the analyses due to the extremely low current smoking rate. The progression rate in the continuously drinking group was 38.7%, whereas that of the sober group stood at 36.2% and the non-drinking group at baseline and drinking group at follow-up at 31.7%. The group that changed from non-exercise to exercise showed the lowest progression rate (22.6%). In terms of FSG measurements, the continuously normal FSG group showed the lowest progression rate (34.9%), and the continuously high FSG group showed the highest progression rate (86.7%). With regards TC measurements, the group with improvements in TC (42.3%) had a lower progression rate than the consistently HTC group (48.4%) (Table 3).

The HRs of progression to HTN are shown by age, family history of CVD, changes in health behavior factors (smoking, alcohol drinking, regular exercise), and changes in comorbidities (BMI, FSG, TC) in prehypertensive subjects at baseline (Table 4). With advanced age, the progression to HTN was accelerated. In addition, compared to the group without a family history of CVD, men with such a background showed about a 1.146 times higher risk of contracting HTN. Improvements in CVD risk factors were also closely associated with a decreased risk of progression to HTN. Improvements in health behaviors and co-morbidities significantly diminished the risk of HTN from 1.269 to 1.531 times in men. In women, smoking was excluded from analyses due to the extremely small size of the sample. Unlike in men, subjects taking regular exercise reduced their probability of HTN by 1.873 times (95% CI: 0.426-0.669).

## DISCUSSION

To our knowledge, this is the first investigation of the relationship between changes in CVD risk factors and the rate of progression of HTN using national longitudinal data in Korea. These longitudinal data showed that improvements in CVD risk factors diminished the rate of progression of HTN in prehypertensive men and women. The periodic screening of BP in adults has been recommended to detect the onset of HTN [3, 16] so that appropriate measures can be taken to prevent the morbidity and mortality associated with raised BP [17]. The European Task Force on Prevention of Coronary Disease recommended a follow-up interval of up to 5 yr [18]. Current recommendations for follow-up BP screening of individuals without HTN are empirical rather than evidence-based, and vary widely [16, 18].

It has previously been reported that older age, higher BMI, and elevated baseline BP are independent risk factors for future HTN [3, 19, 20]. Recent reports from the Framingham Heart Study suggested that persons with normal BP at 55 years of age had a 90% lifetime risk of developing HTN [3, 21]. An increasing incidence of HTN with increasing age was found, which was in line with other results [11, 22]. Cigarette smoking is well established as a causal factor in coronary heart disease and stroke. According to the Korea Medical Insurance Corporation (KMIC) study, smoking is also a major independent risk factor for ischemic heart disease (IHD), CVD, and atherosclerotic cardiovascular disease (ASCVD). The RR from current smoking was reported to be 3.3 (95% CI: 1.7-6.2) for IHD and 1.6 (95% CI: 1.2-2.3) for CVD [23]. In the Cox proportional hazard model, the HR (95% CI) of ex-smokers was 0.756 (0.696-0.821) in men, compared with continuous smokers among the current smokers at baseline. Heavy alcohol consumption may cause other cardiac disorders and is associated with an increased risk of stroke [24]. The HR (95% CI) of non-drinkers was 0.669 (0.607-0.738) in men and 0.829 (0.613-1.121) in women, respectively, compared with continuous drinkers. In general, regular exercise reduces the risk of coronary artery disesase (CHD). Regular exercise has BP-lowering effects [25]. It was reported that individuals participating in 20 min of light or moderate regular exercise every day had about a 30% lower risk of death from CHD than sedentary individuals [26]. In this study, in those not taking regular exercise at baseline, the HR (95% CI) of subjects who later started regular exercise was 0.653 (0.593-0.718) in men and 0.534 (0.426-0.669) in women, compared with those not participating in regular exercise at all. Obesity contributes to elevated BP [17]. It has consistently been proven through clinical trials that reducing weight can lower SBP and DBP [27]. The risk of HTN is higher among obese people (BMI ≥25 kg/m^2^). The BMI is significantly and positively correlated with both SBP and DBP [28]. Jee et al. [15] found that underweight, overweight and obese men and women had higher rates of death than normal weight men and women. Several previous studies have reported that weight loss might play a protective role in the progression to HTN [9, 29, 30]. In this study, the HR (95% CI) of the weight loss group (BMI <25 kg/m^2^) was 0.715 (0.593-0.861) in men and 0.634 (0.401-1.001) in women, compared with the continuously obese group (BMI ≥25 kg/m^2^). Kim et al. [11] reported that diabetes mellitus (DM) was an independent risk factor for HTN in women, but not men. In this study, the baseline FSG level was associated with the progression of HTN in both men and women. However, lowering the FSG did not play a protective role in the progression to HTN in either men or women. In previous studies, a higher TC was a predictor of progression to HTN [30]. In another study, the direct and progressive relationship between TC and CVD was reported [31]. In this study, TC was a strong predictor of the progression to HTN. Compared to the consistently high TC group (TC ≥240 mg/dL), the 'back to normal' TC group (TC <240 mg/dL) lowered their risk by 1.27 times in men, controlling for other CVD risk factors.

This study has several potential limitations. First, there was no information on antihypertensive medications in this population, which could be a confounding factor in the association between the CVD risk factors and BP. The absence of medication information could have resulted in a conservative estimate of the incidence of PreHTN and HTN. Second, misclassification bias could have arisen from measurement error because the biennial health examinations were carried out at various hospitals throughout the country and the laboratory techniques varied. However, the data collected were subject to internal and external quality control [32, 33]. Third, we categorized the individuals based on the average BP in 1994 and 1995 obtained from a single BP measurement, as in the recent study by Hansen et al. [34], while other studies used two readings [2]. Lastly, since we used data from biennial BP measurements, we had no information on possible changes during the intervening period. It is possible that individuals with HTN changed their unhealthy lifestyles. For example, when a current smoker was diagnosed with PreHTN at the first examination, she/he may have subsequently quit smoking. After stopping smoking, BP could have returned to normal. We are not in a position to know whether smoking cessation alone contributed to decreasing BP.

Despite several limitations, this study has a few strengths, including the extensive coverage of South Koreans with repeated measures of BP, FSG, TC, weight, and height through the KCPS data. This large sample size and the prospective design had sufficient statistical power in general, even in subgroup and dose-response analyses [23, 35].

In conclusion, this study confirmed the findings of previous studies demonstrating a relationship between changes in CVD risk factors and the progression from PreHTN to HTN. With regards public health implications in Korea, the prehypertensive population should be targeted for specific health intervention to change their unhealthy lifestyles. Large intervention studies should be established urgently in order to confirm the results of this study.

## Figures and Tables

**Table 1:**
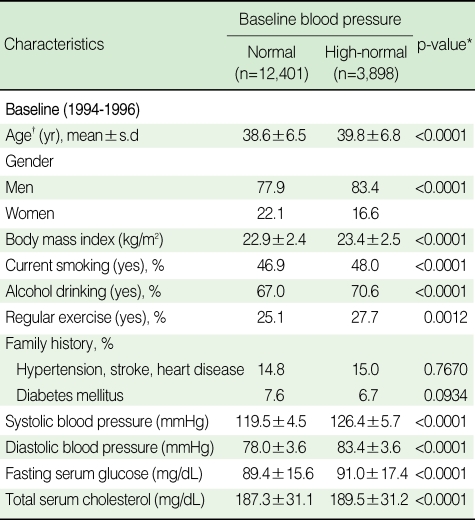
Baseline characteristics of the study population according to baseline blood pressure

^*^p-value by t-test or χ^2^ test; ^†^Age and family history in 1992 presented because the data included individuals who participated in the health exam in 1992 from the KCPS data.

**Table 2:**
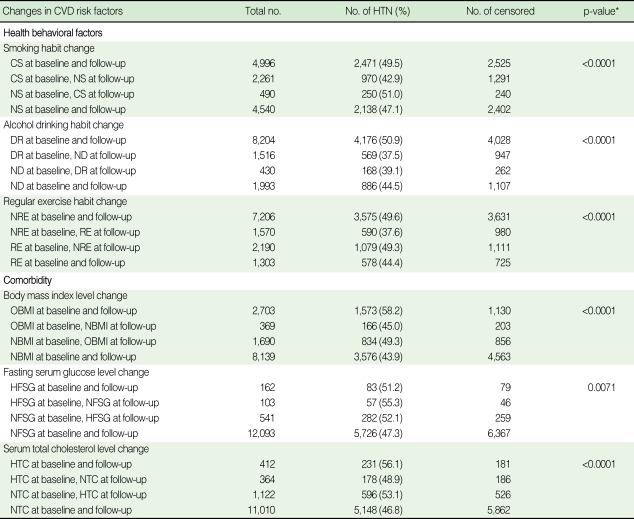
Relationship between changes in cardiovascular disease risk factors and progression to hypertension in prehypertensive men at baseline

CVD, cardiovascular disease; HTN, hypertension; NS, never or former smoker; CS, current smoker; NDR, non-drinker; DR, current drinker; RE, those taking regular exercise; NRE, those not taking regular exercise; NBMI, those with a BMI <25.0 kg/m^2^; OBMI, those with a BMI ≥25.0 kg/m^2^; NFSG, those with a FSG <100 mg/dL; HFSG, those with a FSG ≥126 mg/dL; NTC, those with a TC <240 mg/dL; HTC, those with a TC ≥240 mg/dL. ^*^p-value by log-rank test.

**Table 3:**
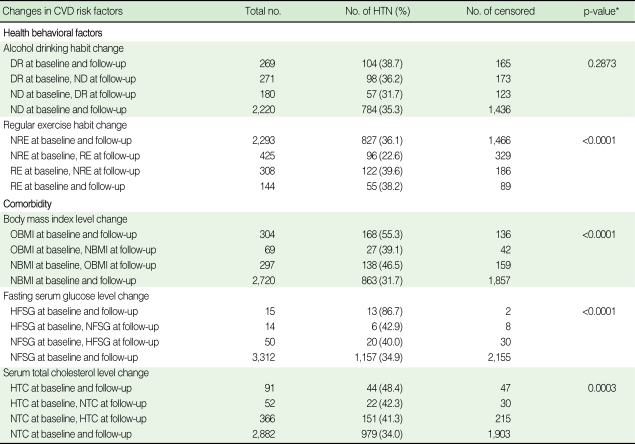
Relationship between changes in cardiovascular disease risk factors and progression to hypertension in prehypertensive women at baseline

CVD, cardiovascular disease; HTN, hypertension; NDR, non-drinker; DR, current drinker; RE, those taking regular exercise; NRE, those not taking regular exercise; NBMI, those with a BMI <25.0 kg/m^2^; OBMI, those with a BMI ≥25.0 kg/m^2^; NFSG, those with a FSG <100 mg/dL; HFSG, those with a FSG ≥126 mg/dL; NTC, those with a TC <240 mg/dl; HTC, those with a TC ≥240 mg/dL. ^*^p-value by log-rank test.

**Table 4:**
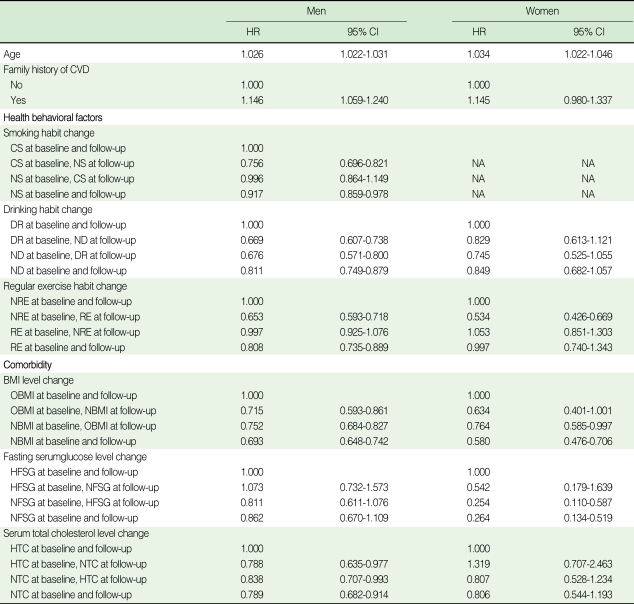
Risk factors for progression to hypertension in prehypertensive group at baseline

All covariates were included in the model. HR, hazard ratio; CI, confidence interval; CVD, cardiovascular disease; HTN, hypertension; NA, not available; NS, never or former smoker; CS, current smoker; NDR, non-drinker; DR, current drinker; RE, those taking regular exercise; NRE, those not taking regular exercise; NBMI, those with a BMI <25.0 kg/m^2^; OBMI, those with a BMI ≥25.0 kg/m^2^; NFSG, those with a FSG <100 mg/dL; HFSG, those with a FSG ≥126 mg/dL; NTC, those with a TC <240 mg/dL; HTC, those with a TC ≥240 mg/dL.

## References

[1] World Health Organization (1993). 1993 guidelines for the management of mild hypertension: memorandum from a World Health Organization/international society of hypertension meeting. Guidelines Subcommittee. J Hypertens.

[2] Vasan RS, Larson MG, Leip EP, Evans JC, O'Donnell CJ, Kannel WB (2001). Impact of high-normal blood pressure on the risk of cardiovascular disease. N Engl J Med.

[3] Chobanian AV, Bakris GL, Black HR, Cushman WC, Green LA, Izzo JL (2003). The Seventh Report of the Joint National Committee on Prevention, Detection, Evaluation, and Treatment of High Blood Pressure: the JNC 7 report. JAMA.

[4] Erdine S, Ari O, Zanchetti A, Cifkova R, Fagard R, Kjeldsen S (2006). ESH-ESC guideline for the management of hypertension. Herz.

[5] Moser M (1977). Detection, evaluation, and treatment of high blood pressure. N Y State J Med.

[6] Leitschuh M, Cupples LA, Kannel W, Gagnon D, Chobanian A (1991). High-normal blood pressure progression to hypertension in the Framingham Heart Study. Hypertension.

[7] Sagie A, Larson MG, Levy D (1993). The natural history of borderline isolated systolic hypertension. N Engl J Med.

[8] Vasan RS, Larson MG, Leip EP, Kannel WB, Levy D (2001). Assessment of frequency of progression to hypertension in non-hypertensive participants in the Framingham Heart Study: a cohort study. Lancet.

[9] Winegarden CR (2005). From "prehypertension" to hypertension?: Additional evidence. Ann Epidemiol.

[10] Zhang H, Thijs L, Kuznetsova T, Fagard RH, Li X, Staessen JA (2006). Progression to hypertension in the non-hypertensive participants in the Flemish Study on Environment, Genes and Health Outcomes. J Hypertens.

[11] Kim J, Kim E, Yi H, Joo S, Shin K, Kim J (2006). Short-term incidence rate of hypertension in Korea middle-aged adults. J Hypertens.

[12] Chien KL, Hsu HC, Sung FC, Su TC, Chen MF, Lee YT (2007). Incidence of hypertension and risk of cardiovascular events among ethnic Chinese: report from a community-based cohort study in Taiwan. J Hypertens.

[13] Lee JH, Hwang SY, Kim EJ, Kim MJ (2006). Comparison of Risk factors between prehypertension and hypertension in Korean male industrial workers. Public Health Nurs.

[14] Jee SH, Samet JM, Ohrr H, Kim JH, Kim IS (2004). Smoking and cancer risk in Korean men and women. Cancer Causes Control.

[15] Jee SH, Sull JW, Park J, Lee SY, Ohrr H, Guallar E (2006). Body-mass index and mortality in Korean men and women. N Engl J Med.

[16] (1997). The sixth report of the Joint National Committee on prevention, detection, evaluation, and treatment of high blood pressure. Arch Intern med.

[17] Stamler J, Stamler R, Neaton JD (1993). Blood pressure, systolic and diastolic, and cardiovascular risks. US population data. Arch Intern Med.

[18] Wood D, De Backer G, Faergeman O, Graham I, Mancia G, Pyörälä K (1998). Prevention of coronary heart disease in clinical practice. Summary of recommendations of the Second Joint Task Force of European and other Societies on Coronary Prevention. J Hypertens.

[19] Whelton PK, He J, Appel LJ, Cutler JA, Havas S, Kotchen TA (2002). Primary prevention of hypertension: clinical and public health advisory from The National High Blood Pressure Education Program. JAMA.

[20] Lee JS, Kawakubu K, Kashihara H, Mori K (2004). Effect of long-term body weight change on the incidence of hypertension in Japanese men and women. Int J Obes Relat Metab Disord.

[21] Vasan RS, Beiser A, Seshadri S, Larson MG, Kannel WB, D'Agostino RB (2002). Residual lifetime risk for developing hypertension in middle-aged women and men: The Framingham Heart Study. JAMA.

[22] Jee SH, Appel LJ, Suh I, Whelton PK, Kim IS (1998). Prevalence of cardiovascular risk factors in South Korean adults: results from the Korea Medical Insurance Corporation (KMIC) Study. Ann Epidemiol.

[23] Jee SH, Suh I, Kim IS, Appel LJ (1999). Smoking and atherosclerotic cardiovascular disease in men with low levels of serum cholesterol: the Korea Medical Insurance Corporation Study. JAMA.

[24] Wannamethee SG, Shaper AG (1996). Patterns of alcohol intake and risk of stroke in middle-aged British men. Stroke.

[25] Arakawa K (1996). Effect of exercise on hypertension and associated complications. Hypertens Res.

[26] Leon AS, Myers MJ, Connett J (1997). Leisure time physical activity and the 16-year risks of mortality from coronary heart disease and all-causes in the Multiple Risk Factor Intervention Trial (MRFIT). Int J Sports Med.

[27] (1992). The effects of nonphamacologic interventions on blood pressure of persons with high normal levels. Results of the Trials of Hypertension Prevention, Phase 1. JAMA.

[28] Tesfaye F, Nawi NG, Van Minh H, Byass P, Berhane Y, Bonita R (2007). Association between body mass index and blood pressure across three populations in Africa and Asia. J Hum Hypertens.

[29] Franklin SS, Pio JR, Wong ND, Larson MG, Leip EP, Vasan RS (2005). Predictors of new-onset diastolic and systolic hypertension: the Framingham Heart Study. Circulation.

[30] de Simone G, Devereux RB, Chinali M, Roman MJ, Best LG, Welty TK (2006). Risk factors for arterial hypertension in adults with initial optimal blood pressure: the Strong Heart Study. Hypertension.

[31] Neaton JD, Wentworth D, Multiple Risk Factor Intervention Trial Research Group (1992). Serum cholesterol, blood pressure, cigarette smoking, and death from coronary heart disease. Overall findings and difference by age for 316,099 white men. Arch Intern Med.

[32] Chung RM, Kim SH, Kim YS, Kim YK, Kim JQ, Yi KN (1991). Annual report on external quality assessment in clinical chemistry in Korea. J Clin Pathol Qual Control.

[33] Min WK, Kim YK, Kwon OH, Kim KD, Kim SS, Kim JW (2001). Annual report on external quality assessment in clinical chemistry in Korea (2000). J Clin Pathol Qual Control.

[34] Hansen TW, Staessen JA, Zhang H, Torp-Pedersen D, Rasmussen S, Thijs L (2007). Cardiovascular outcome in relation to progression to hypertension in the Copenhagen MONICA Cohort. Am J Hypertens.

[35] Yun JE (2007). The relation between weight changes and alanine aminotransferase levels in a nonalcoholic population [dissertation].

